# Authentic Leadership and Nurses’ Innovative Behaviour: A Two‐Wave Multicentre Study of Psychological Empowerment and Leader–Member Exchange

**DOI:** 10.1155/jonm/7337513

**Published:** 2026-08-30

**Authors:** Gui Yu, Jing Fang, Xiaojuan Yang, Lijuan Yin, Yanli Zeng

**Affiliations:** ^1^ Department of Urology, Sichuan Academy of Medical Sciences and Sichuan Provincial People’s Hospital, Chengdu, Sichuan, China, scu.edu.cn; ^2^ College of Nursing, Chengdu University of Traditional Chinese Medicine, Chengdu, China, cdutcm.edu.cn; ^3^ Department of Breast Surgery, Sichuan Academy of Medical Sciences and Sichuan Provincial People’s Hospital, Chengdu, Sichuan, China, scu.edu.cn; ^4^ Department of Nephrology, Sichuan Academy of Medical Sciences and Sichuan Provincial People’s Hospital, Chengdu, Sichuan, China, scu.edu.cn

**Keywords:** Authentic leadership, innovative behaviour, leader–member exchange, nurses, psychological empowerment

## Abstract

**Aim:**

To examine the association between authentic leadership and nurses’ innovative behaviour and to test the parallel mediating roles of psychological empowerment and leader–member exchange.

**Design:**

A two‐wave, time‐lagged, multicentre survey design.

**Methods:**

Clinical nurses from 12 public hospitals across eastern, central and western China participated. A total of 2291 nurses completed the baseline survey, and 1812 completed the follow‐up assessment and were included in the final analysis, yielding a retention rate of 79.1%. Authentic leadership, psychological empowerment and leader–member exchange were measured at Time 1, and nurses’ innovative behaviour was measured at Time 2. Structural equation modelling and bootstrap analysis were conducted to test the hypothesised mediation model.

**Results:**

Authentic leadership was positively associated with psychological empowerment and leader–member exchange. Both mediators were positively associated with nurses’ innovative behaviour. The direct association between authentic leadership and innovative behaviour was not significant after the mediators were included. Psychological empowerment demonstrated a stronger mediating effect than leader–member exchange.

**Conclusion:**

Authentic leadership may support nurses’ innovative behaviour primarily by strengthening psychological empowerment, with leader–member exchange providing an additional relational pathway.

**Implications for the Profession:**

Nurse managers should develop authentic leadership practices that strengthen nurses’ psychological resources and workplace relationships to foster innovation in clinical settings.

**Reporting Method:**

This study adhered to STROBE reporting guidelines.

## 1. Introduction

Innovation has become an essential capability for nurses working in increasingly complex and resource‐constrained healthcare systems. As frontline professionals, nurses are expected to adapt clinical processes, generate novel solutions and contribute to continuous quality improvement to enhance patient care and organisational performance [1]. Accordingly, nurses’ innovative behaviour (IB), defined as the generation, promotion and implementation of new ideas in clinical practice, has emerged as a critical competency in contemporary nursing [2, 3]. Identifying organisational and interpersonal conditions that foster such behaviour has, therefore, become an important concern in nursing management research.

Leadership plays a pivotal role in shaping nurses’ motivation, psychological experiences and behavioural responses. Among various leadership approaches, authentic leadership (AL) has attracted increasing attention because of its emphasis on self‐awareness, relational transparency, balanced processing and an internalised moral perspective [4]. Emerging evidence suggests that AL enhances trust, engagement and creativity among nurses, thereby creating conditions conducive to innovation. Notably, a recent scoping review synthesising global evidence reported a positive association between AL and nurses’ innovative and creative behaviours across diverse healthcare contexts [5]. Despite this growing interest, the mechanisms through which AL translates into IB remain insufficiently understood.

Theoretical perspectives suggest that leadership may influence IB through multiple pathways. From a cognitive–motivational perspective, psychological empowerment (PE) reflects employees’ perceptions of meaning, competence, self‐determination and impact [6] and has been associated with proactive and innovation‐related behaviours among nurses [3, 7]. From a relational perspective, leader–member exchange (LMX) represents the quality of reciprocal relationships between leaders and followers and has long been recognised as a predictor of discretionary work behaviours, creativity and innovation [8, 9]. However, previous studies have typically examined these mechanisms independently, focused primarily on organisational climate rather than leadership or relied on analytical approaches that were unable to simultaneously examine multiple pathways.

Recent reviews have highlighted substantial heterogeneity in theoretical framing and methodological approaches within the nursing innovation literature, underscoring the need for more integrative and theory‐driven models [5]. In particular, few studies have simultaneously examined PE and LMX as parallel mechanisms linking AL to nurses’ IB. Such integration is theoretically warranted. Social information processing theory (SIPT) explains how leaders provide contextual cues that shape employees’ psychological interpretations and motivational states [10], whereas social exchange theory (SET) explains how leadership behaviours foster reciprocal relationships that encourage discretionary behaviours [11]. Together, these perspectives suggest that AL may simultaneously activate cognitive–motivational and relational–exchange mechanisms that support innovation.

Furthermore, most previous studies have relied on cross‐sectional designs, limiting understanding of how leadership‐related perceptions and innovation‐related outcomes may unfold over time. Greater attention to temporally separated designs may help strengthen inferences regarding the processes through which leadership influences innovation.

To address these gaps, the present study integrates social information processing theory and SET to examine how AL influences nurses’ IB through complementary cognitive–motivational and relational mechanisms. IB is a complex discretionary behaviour that requires both intrinsic motivation and a willingness to invest effort beyond formal role expectations. SIPT suggests that authentic leaders provide informational cues that shape nurses’ perceptions of meaning, competence, autonomy and impact, thereby enhancing PE. In contrast, SET suggests that authentic leaders foster high‐quality leader–member relationships characterised by trust, support and reciprocity, which encourage nurses to engage in discretionary behaviours that benefit the organisation. Examining these mechanisms simultaneously may therefore provide a more comprehensive explanation of how AL promotes IB than either perspective alone. Using a multicentre two‐wave survey design and structural equation modelling (SEM), this study investigates the parallel mediating roles of PE and LMX in the relationship between AL and nurses’ IB.

## 2. Theoretical Framework and Hypotheses Development

### 2.1. Theoretical Framework

AL may influence nurses’ IB through multiple psychological and relational processes. However, existing studies have typically focused on isolated mechanisms, making it difficult to explain how leadership perceptions are translated into innovation‐related actions. To address this limitation, the present study integrates SIPT and SET as complementary perspectives. SIPT explains how employees interpret leadership cues and develop cognitive and motivational responses, whereas SET explains how leadership behaviours shape reciprocal relationships and discretionary behavioural exchanges. Together, these theories provide a coherent framework for understanding how AL may influence IB through both empowerment‐based and exchange‐based mechanisms.

#### 2.1.1. SIPT

SIPT proposes that employees interpret cues from their social environment to construct perceptions of their work and guide behavioural responses [10]. Authentic leaders provide salient informational cues through transparency, ethical consistency, and balanced decision‐making. These cues signal trust, autonomy and the value of employees’ contributions, thereby shaping how nurses perceive their work environment.

One important outcome of this interpretive process is PE, defined as perceptions of meaning, competence, self‐determination and impact [6]. Nurses who perceive greater empowerment are more likely to take initiative, engage in proactive problem‐solving and pursue innovative ideas. Previous studies have linked AL to higher levels of PE and have shown that empowered nurses are more likely to engage in innovation‐related behaviours [7, 12]. Accordingly, SIPT provides a useful framework for explaining how AL may influence IB through cognitive–motivational processes.

#### 2.1.2. SET

SET offers a complementary explanation by emphasising the relational consequences of leadership behaviours [11]. According to SET, supportive and trustworthy leaders foster reciprocal relationships characterised by mutual respect, trust and obligation. Authentic leaders are particularly well positioned to develop such relationships because they demonstrate relational transparency, fairness and ethical consistency.

LMX reflects the quality of these reciprocal relationships [8]. High‐quality LMX relationships provide nurses with support, recognition, and access to resources, which may encourage them to reciprocate through discretionary behaviours that benefit the organisation. IB is often regarded as one such extra‐role behaviour because it requires employees to invest effort beyond formal job requirements. Therefore, SET suggests that AL may promote IB by strengthening LMX relationships.

#### 2.1.3. Integrating SIPT and SET: A Parallel Mediation Framework

Taken together, PE and LMX represent two theoretically distinct yet complementary mechanisms through which AL may influence nurses’ IB. PE reflects a cognitive–motivational pathway, consistent with SIPT, through which leaders shape nurses’ internal interpretations and intrinsic motivation. In contrast, LMX reflects a relational–exchange pathway, consistent with SET, through which leaders shape reciprocal obligations and discretionary behavioural responses.

Despite their conceptual complementarity, prior studies have typically examined these mechanisms in isolation or relied on analytic approaches that cannot simultaneously account for measurement error and multiple mediation pathways. Recent theoretical and empirical work has, therefore, called for more integrative, mechanism‐rich models to clarify how AL translates into IB [5, 13].

**FIGURE 1:**
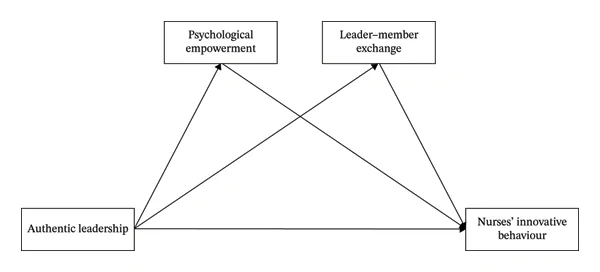
Hypothesised parallel mediation model of authentic leadership, psychological empowerment, leader–member exchange and nurses’ innovative behaviour.

Responding to these gaps, the present study proposes a parallel mediation model in which PE and LMX jointly mediate the association between AL and nurses’ IB. The proposed conceptual framework is illustrated in Figure 1.

### 2.2. Hypotheses Development

Consistent with prior leadership and innovation research, the direct relationship between AL and IB was retained in the conceptual model. Testing this direct association allows examination of whether AL exerts a direct influence on IB or whether its effects are primarily transmitted through PE and LMX.

#### 2.2.1. AL and Psychological Empowerment

According to SIPT, employees interpret cues from their work environment to construct perceptions of their roles, responsibilities and opportunities for action [10]. Authentic leaders communicate openly, demonstrate consistency between values and actions and encourage balanced decision‐making [4]. These behaviours provide informational cues that signal trust, autonomy and respect, thereby shaping employees’ perceptions of their work environment.

PE reflects employees’ perceptions of meaning, competence, self‐determination and impact [6]. Previous studies have shown that supportive leadership behaviours enhance employees’ sense of empowerment by increasing autonomy, confidence and participation in decision‐making [12, 14]. In nursing settings, AL has been associated with higher levels of PE and positive work‐related outcomes [7, 15].

Therefore, the following hypothesis was proposed:

 H1. AL is positively associated with PE.

#### 2.2.2. AL and LMX

SET suggests that supportive and trustworthy leadership promotes reciprocal relationships characterised by mutual respect, trust and obligation [11]. Authentic leaders demonstrate relational transparency and ethical consistency, which facilitate the development of high‐quality exchanges with followers [4].

LMX reflects the quality of the relationship between leaders and followers and is characterised by trust, support and mutual commitment [8]. Previous research indicates that AL is positively associated with high‐quality LMX relationships because authentic leaders are perceived as trustworthy, fair and supportive [16, 17].

Accordingly, we hypothesised that

 H2. AL is positively associated with LMX.

#### 2.2.3. AL and IB

Leadership has long been recognised as an important antecedent of IB because leaders influence employees’ motivation, access to resources and willingness to engage in change‐oriented activities. Authentic leaders create work environments characterised by openness, trust and ethical consistency, which may encourage employees to express new ideas and challenge existing practices [4].

Previous studies have reported positive associations between AL and innovation‐related outcomes among nurses and other professional groups [5, 13]. However, the mechanisms underlying this relationship remain insufficiently understood. Some scholars have suggested that the influence of AL on IB may operate primarily through psychological and relational processes rather than through a direct effect.

Consistent with prior research, the direct association between AL and IB was retained in the conceptual model. Testing this pathway allows examination of whether AL exerts a direct influence on nurses’ IB or whether its effects are transmitted primarily through PE and LMX.

Therefore, the following hypothesis was proposed:

 H3. AL is positively associated with nurses’ IB.

#### 2.2.4. Psychological Empowerment and IB

PE represents an intrinsic motivational state characterised by perceptions of meaning, competence, self‐determination and impact [6]. According to SIPT, employees’ interpretations of their work environment influence their motivation and behavioural responses [10]. When nurses perceive themselves as empowered, they are more likely to feel confident in their abilities, take initiative and engage in proactive behaviours that extend beyond routine job requirements.

IB inherently involves uncertainty, risk‐taking and the willingness to challenge existing practices. Nurses who experience higher levels of PE may be more willing to identify problems, generate novel solutions and implement improvements because they perceive themselves as capable of influencing clinical outcomes. Previous studies have consistently demonstrated positive associations between PE and innovation‐related outcomes among nurses and healthcare professionals [3, 7].

Accordingly, the following hypothesis was proposed:

 H4. PE is positively associated with nurses’ IB.

#### 2.2.5. LMX and IB

LMX reflects the quality of reciprocal relationships between leaders and followers and is characterised by mutual trust, support and respect [8]. SET proposes that employees who experience supportive and beneficial relationships with their leaders are likely to reciprocate through positive attitudes and discretionary behaviours that benefit the organisation [11].

IB is often considered an extra‐role behaviour because it requires employees to invest additional effort beyond formal job responsibilities. Nurses who experience high‐quality LMX relationships may be more willing to contribute innovative ideas and participate in improvement initiatives because they perceive organisational support and trust from their leaders. Previous research has reported positive associations between LMX and IB across healthcare and organisational settings [9, 17].

Therefore, we hypothesised that

 H5. LMX is positively associated with nurses’ IB.

#### 2.2.6. Mediating Roles of Psychological Empowerment and LMX

Building on social information processing theory and SET, the present study proposes that AL may influence nurses’ IB through both cognitive–motivational and relational–exchange mechanisms.

From the perspective of SIPT, authentic leaders provide positive informational cues that enhance nurses’ perceptions of meaning, competence, autonomy and impact, thereby increasing PE. Empowered nurses are more likely to engage in proactive and innovation‐related behaviours because they perceive themselves as capable of initiating and implementing change.

From the perspective of SET, authentic leaders foster trusting and supportive relationships with nurses, resulting in high‐quality LMX relationships. These relationships create reciprocal obligations that encourage employees to contribute beyond formal role requirements, including through IB.

Although PE and LMX are conceptually distinct, they are not mutually exclusive. AL may simultaneously shape how nurses interpret their work environment and how they relate to their leaders. Consequently, both empowerment‐based and exchange‐based mechanisms may operate concurrently to facilitate IB.

Therefore, the following hypotheses were proposed:

 H6. PE mediates the association between AL and nurses’ IB.
 H7. LMX mediates the association between AL and nurses’ IB.
 H8. PE and LMX jointly mediate the association between AL and nurses’ IB.

## 3. Methods

### 3.1. Study Design and Participants

A two‐wave, time‐lagged, multicentre survey design was employed. Clinical nurses were recruited from 12 public hospitals located in eastern, central and western China using convenience sampling. Inclusion criteria were registered nurses who had at least 1 year of clinical experience in their current hospital and provided informed consent. Exclusion criteria included nurses who were not directly involved in patient care during the survey period (e.g., due to sick or personal leave) or who held managerial positions at or above the head‐nurse level.

The two‐wave design was adopted to introduce temporal separation between predictor and outcome variables and to reduce the risk of common method bias. Consistent with previous leadership and organisational behaviour research, a 1‐month interval was selected because perceptions of leadership, empowerment and LMX are relatively stable over short periods, whereas innovation‐related behaviours may emerge subsequently as employees translate these perceptions into workplace actions. Although a two‐wave design cannot establish definitive causality, it provides stronger temporal ordering and methodological rigour than a single cross‐sectional survey.

### 3.2. Common Method Bias Control and Data Collection

To reduce the risk of common method bias, data were collected at two time points separated by a 1‐month interval [18]. At Time 1, participants reported demographic characteristics, AL, PE, and LMX. At Time 2, they reported nurses’ IB.

Instructional manipulation checks (IMCs) were embedded in both surveys to enhance response quality [19]. Only participants who passed all attention checks were retained. Surveys were administered via Credamo, a secure online platform that enabled automatic matching of responses across waves using anonymous identification codes. Data collection took place between March 1 and June 30, 2025.

### 3.3. Sample Matching and Final Sample

A total of 2291 nurses completed the Time 1 survey. Among them, 1939 nurses also completed the Time 2 survey. Responses were matched using unique anonymous identification codes generated by the survey platform. After excluding unmatched responses, identification irregularities, and failed IMCs, the final matched sample consisted of 1812 nurses, representing a retention rate of 79.1%. During the matching process, 41 cases were excluded because responses could not be reliably matched across the two waves due to missing or inconsistent identifiers. An additional 19 cases were removed due to identification irregularities, including duplicate entries, invalid identifiers, or internally inconsistent codes.

To ensure data validity, 67 cases were further excluded for failing one or more IMCs. After these exclusions (total excluded = 127 cases), the final matched sample consisted of 1812 nurses, which was used for all subsequent analyses.

### 3.4. Measures

All constructs were assessed using validated Chinese versions of established instruments.

#### 3.4.1. AL

AL was measured using the 16‐item AL Questionnaire developed by Walumbwa et al. [4] and validated in Chinese by Zhang and Zheng [20]. The scale comprises four dimensions: self‐awareness, relational transparency, balanced processing, and internalised moral perspective. Items are rated on a 5‐point scale ranging from 0 (‘*not at all*’) to 4 (‘*frequently, if not always*’), with higher scores indicating stronger perceptions of leader authenticity.

#### 3.4.2. PE

PE was assessed using Spreitzer’s 12‐item scale [6], validated in Chinese by Sun et al. [21]. The scale includes four dimensions: meaning, competence, self‐determination and impact. Responses are rated from 1 (‘*strongly disagree*’) to 5 (‘*strongly agree*’).

#### 3.4.3. LMX

LMX was measured using the Chinese revised version of the LMX scale [22], adapted from Liden [23]. The scale consists of 16 items across four dimensions: affect, loyalty, contribution and professional respect. Items were rated on a 5‐point Likert scale ranging from 1 (‘*strongly disagree*’) to 5 (‘*strongly agree*’), with higher scores indicating higher quality LMX relationships.

#### 3.4.4. Nurses’ IB

Nurses’ IB was assessed using the Chinese version of the Nurse Innovative Behaviour Scale [24], which includes 10 items covering idea generation, idea promotion/receiving support, and idea realisation. Items are rated from 1 (“never”) to 5 (“always”).

Internal consistency was excellent for all scales in the present study, with Cronbach’s *α* coefficients ranging from 0.951 to 0.986 (Table 1).

**TABLE 1:** Means, standard deviations, reliability, validity and correlations among study variables (*N* = 1812).

Variable	M	SD	*α*	CR	AVE	1	2	3	4
1 AL	4.39	0.78	0.983	0.968	0.883	0.940			
2 PE	4.20	0.68	0.951	0.897	0.689	0.608^∗∗^	0.830		
3 LMX	4.31	0.78	0.986	0.956	0.844	0.735^∗∗^	0.708^∗∗^	0.919	
4 IB	3.92	0.89	0.970	0.940	0.839	0.510^∗∗^	0.646^∗∗^	0.567^∗∗^	0.916

*Note:* IB = nurses’ innovative behaviour; square roots of AVE are presented on the diagonal. All correlations were statistically significant at ^∗∗^ *p* < 0.01.

Abbreviations: AL, authentic leadership; AVE, average variance extracted; CR, composite reliability; LMX, leader–member exchange; PE, psychological empowerment.

### 3.5. Data Analysis

Data analyses were conducted using SPSS version 26.0 and AMOS version 24.0. Descriptive statistics and Pearson correlation coefficients were first computed to examine distributions and bivariate associations among study variables.

Confirmatory factor analysis (CFA) was performed to evaluate construct validity. For AL, PE, and LMX, second‐order CFA models were specified, with theoretically defined first‐order dimensions loading onto a higher order latent construct. A four‐factor measurement model including all study variables was then tested to assess discriminant validity. Model fit was evaluated using the chi‐square statistic (*χ* ^2^), comparative fit index (CFI), Tucker–Lewis index (TLI), and root mean square error of approximation (RMSEA), with CFI and TLI values ≥ 0.90 and RMSEA values ≤ 0.08 indicating acceptable fit [25].

In addition to Cronbach’s *α*, composite reliability (CR) was calculated to assess internal consistency. Convergent validity was evaluated using average variance extracted (AVE), with values ≥ 0.50 indicating adequate convergence. Discriminant validity was assessed using the Fornell–Larcker criterion.

To examine potential common method variance, a single‐factor CFA was conducted in which all measurement items loaded onto one latent factor. The poor fit of this model (Table 2) suggested that common method bias was unlikely to substantially account for the observed relationships [26].

**TABLE 2:** Confirmatory factor analysis: comparison of competing measurement models.

Model	*χ* ^2^	df	*χ* ^2^/df	CFI	TLI	RMSEA
Hypothesised four‐factor model (AL, PE, LMX and IB)	1110.33	84	13.22	0.968	0.960	0.082
Three‐factor model (PE + LMX combined)	3310.29	87	38.05	0.900	0.880	0.143
Three‐factor model (PE + IB combined)	4440.21	87	51.04	0.865	0.838	0.166
Three‐factor model (LMX + IB combined)	5330.17	87	61.27	0.838	0.804	0.182
One‐factor model	11682.10	90	129.80	0.642	0.582	0.267

*Note:* IB = nurses’ innovative behaviour. The hypothesised four‐factor model specified AL, PE, LMX and IB as distinct latent constructs. In the alternative three‐factor models, two theoretically related constructs were combined into a single latent factor. The one‐factor model constrained all indicators to load on a single latent factor and was used to assess discriminant validity and potential common method variance.

Abbreviations: AL, authentic leadership; LMX, leader–member exchange; PE, psychological empowerment.

SEM was used to test the hypothesised relationships, with AL specified as the exogenous variable, PE and LMX as parallel mediators, and nurses’ IB as the outcome variable. Consistent with the theoretical framework, the direct path from AL to IB was initially retained and estimated alongside the indirect pathways. This approach allowed examination of whether the influence of AL on IB was direct, indirect or fully mediated through PE and LMX.

Indirect effects were tested using bootstrapping with 5000 resamples. Bias‐corrected 95% confidence intervals were generated, and mediation was considered statistically significant when the confidence interval did not include zero [27]. Preliminary analyses were conducted to examine whether AL, PE and LMX differed according to demographic characteristics. Independent‐sample *t*‐tests were used for comparisons involving two groups, whereas one‐way analyses of variance was performed for variables with multiple categories.

Variables demonstrating significant differences were considered potential confounding factors. To provide a conservative estimation of the hypothesised relationships, gender, age group, education level, work experience and hospital level were included as covariates in the final SEM.

Because the study employed a time‐lagged design rather than an experimental design, the findings should be interpreted as evidence of theoretically consistent temporal associations rather than definitive causal relationships.

### 3.6. Ethical Considerations

Ethical approval was obtained from the Ethics Review Committee of the Hospital of Chengdu University of Traditional Chinese Medicine (approval no. 2024KL‐146). Written informed consent was obtained from all participants prior to data collection. Participants were assured of confidentiality, anonymity and voluntary participation. All data were anonymised, stored in encrypted, password‐protected files and will be securely retained for 5 years in accordance with institutional policy and international ethical standards.

## 4. Results

### 4.1. Participant Characteristics

A total of 1812 nurses participated in the study. The majority were female (*n* = 1,697, 93.7%), with 115 male nurses (6.6%). Most participants were aged 31–40 years (*n* = 923, 50.8%), followed by those younger than 30 years (*n* = 546, 30.0%) and older than 40 years (*n* = 348, 19.2%).

Most nurses held a bachelor’s degree (*n* = 1,491, 82.1%), while 14.3% (*n* = 260) had a diploma and 3.6% (*n* = 66) held a master’s degree or above. Over half of the sample had more than 10 years of clinical experience (*n* = 952, 52.4%), with 22.2% (*n* = 403) reporting 5–10 years and 25.4% (*n* = 462) less than 5 years of experience.

Differences in the study variables according to demographic characteristics are presented in Supporting Table S1. Significant differences were observed across age groups for AL, PE, and LMX. Work experience was significantly associated with AL and LMX. In contrast, no significant differences were observed according to gender, education level or hospital level. Based on these findings, demographic characteristics were considered in subsequent SEM analyses.

### 4.2. Descriptive Statistics and Correlations

Table 1 presents the means, standard deviations, reliabilities and correlations among the study variables. All scales demonstrated excellent internal consistency (Cronbach’s *α* = 0.951–0.986). AL was positively correlated with PE (*r* = 0.608, *p* < 0.01), LMX (*r* = 0.735, *p* < 0.01) and nurses’ IB (*r* = 0.510, *p* < 0.01). PE was positively associated with LMX (*r* = 0.708, *p* < 0.01) and IB (*r* = 0.646, *p* < 0.01), and LMX was also positively related to IB (*r* = 0.567, *p* < 0.01).

All correlations were in the expected directions and below conventional thresholds for multicollinearity, supporting subsequent SEM analyses.

### 4.3. Measurement Model

All constructs demonstrated satisfactory CR and convergent validity, with CR values exceeding 0.70 and AVE values above 0.50. Discriminant validity was supported, as the square roots of AVE exceeded the interconstruct correlations (Table 1).

The hypothesised four‐factor model demonstrated substantially better fit than all alternative models. As shown in Table 2, the hypothesised four‐factor model (AL, PE, LMX and nurses’ IB) demonstrated acceptable fit (*χ* ^2^ = 1110.33, df = 84, *χ* ^2^/df = 13.22, CFI = 0.968, TLI = 0.960, RMSEA = 0.082). Competing three‐factor models showed substantially poorer fit (CFI = 0.838–0.900; RMSEA = 0.143–0.182), while the one‐factor model exhibited the poorest fit (*χ* ^2^/df = 129.80, CFI = 0.642, RMSEA = 0.267).

The poor fit of the one‐factor model further suggests that common method variance was unlikely to account for the observed relationships.

### 4.4. Structural Model and Hypothesis Testing

A structural model including both direct and indirect paths was tested. The model demonstrated acceptable fit (*χ* ^2^ = 1603.80, df = 150, *χ* ^2^/df = 10.69, CFI = 0.960, TLI = 0.949, RMSEA = 0.073).

**FIGURE 2:**
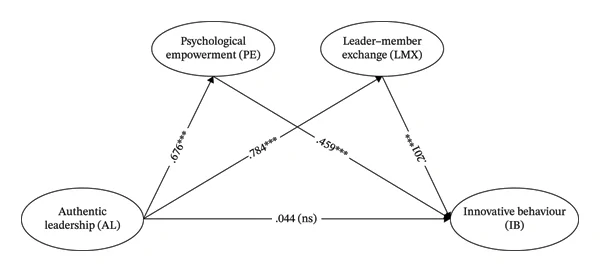
Structural model of authentic leadership, psychological empowerment, leader–member exchange and nurses’ innovative behaviour. Note. Standardised path coefficients are presented; ^∗∗∗^ *p* < 0.001; ns = nonsignificant; *R* ^2^ = 0.422. The direct path from authentic leadership to nurses’ innovative behaviour was not statistically significant; control variables (gender, age, education, work experience and hospital level) were included in the analysis but are omitted from the figure for clarity.

As shown in Figure 2 and Table 3, AL was positively associated with PE (*β* = 0.676, *p* < 0.001) and LMX (*β* = 0.784, *p* < 0.001). PE (*β* = 0.459, *p* < 0.001) and LMX (*β* = 0.201, *p* < 0.001) were positively associated with nurses’ IB.

**TABLE 3:** Structural model results.

Hypothesis	Path	*β*	*B*	SE	95% BC CI for B	*p*
H1	AL ⟶ PE	0.676	0.606	0.034	[0.540, 0.672]	< 0.001
H2	AL ⟶ LMX	0.784	0.813	0.043	[0.730, 0.899]	< 0.001
H3	AL ⟶ IB	0.044	0.041	0.033	[‐0.022, 0.106]	0.206
H4	PE ⟶ IB	0.459	0.472	0.037	[0.399, 0.544]	< 0.001
H5	LMX ⟶ IB	0.201	0.178	0.034	[0.111, 0.243]	< 0.001

*Note: B* = unstandardised coefficient. SE = bootstrap standard error. *β* = standardised coefficient.

Abbreviations: AL, authentic leadership; BC CI, bias‐corrected confidence interval; IB, innovative behaviour; LMX, leader–member exchange; PE, psychological empowerment.

The direct effect of AL on nurses’ IB was not statistically significant (*β* = 0.044, 95% BC CI [−0.024, 0.113], *p* = 0.206), indicating that AL did not directly influence nurses’ IB after PE and LMX were taken into account. Together, the model explained 42.2% of the variance in nurses’ IB.

### 4.5. Mediation Effects

Bootstrapping with 5000 resamples was used to examine the indirect effects (Table 4). The total indirect effect of AL on nurses’ IB was statistically significant (*β* = 0.468, 95% BC CI [0.409, 0.532], *p* < 0.001). The total effect was also significant (*β* = 0.513, 95% BC CI [0.467, 0.556], *p* < 0.001). However, the direct effect of AL on nurses’ IB was not significant (*β* = 0.044, 95% BC CI [−0.024, 0.113], *p* = 0.206). These findings indicate that the relationship between AL and nurses’ IB was fully mediated by PE and LMX. In other words, AL appeared to influence IB indirectly through enhancing nurses’ psychological resources and the quality of leader–member relationships rather than through a direct effect on innovation.

**TABLE 4:** Bootstrap analysis of mediation effects.

Effect	*B*	*β*	Boot SE	95% BC CI	*p*
Direct effect	0.041	0.044	0.033	[−0.022, 0.106]	0.206
Indirect effect via psychological empowerment	0.286	0.310	0.027	[0.236, 0.341]	< 0.001
Indirect effect via leader–member exchange	0.145	0.158	0.027	[0.093, 0.198]	< 0.001
Total indirect effect	0.431	0.468	0.031	[0.373, 0.495]	< 0.001
Total effect	0.471	0.513	0.031	[0.413, 0.531]	< 0.001

**Comparison of indirect effects**	**Difference**	—	**Boot SE**	**95% BC CI**	**p**

Psychological empowerment vs. leader–member exchange	0.140	—	0.044	[0.058, 0.229]	0.002

*Note: B* = unstandardised effect; *β* = standardised effect; Boot SE = bootstrap standard error; BC CI = bias‐corrected confidence interval based on 5000 bootstrap samples. The indirect effect through psychological empowerment represented approximately 60.6% of the total effect, whereas the indirect effect through leader–member exchange represented approximately 30.8% of the total effect. The direct effect was not statistically significant.

Both indirect pathways were statistically significant. The indirect effect through PE (*β* = 0.310) was larger than the indirect effect through LMX (*β* = 0.158). Furthermore, the difference between the two indirect effects was statistically significant (difference = 0.140, 95% BC CI [0.058, 0.229], *p* = 0.002). PE accounted for approximately 60.6% of the total effect, whereas LMX accounted for 30.8%. These findings suggest that AL promotes nurses’ IB primarily by enhancing nurses’ sense of meaning, competence, self‐determination and impact, with LMX serving as a complementary relational mechanism.

## 5. Discussion

### 5.1. Summary of Key Findings

This two‐wave multicentre study examined how AL is associated with nurses’ IB by integrating SIPT and SET within a parallel mediation framework. The findings supported the proposed dual‐pathway model. AL was positively associated with both PE and LMX, and both mediators were positively associated with nurses’ IB.

A key finding was that the direct association between AL and IB was not significant after PE and LMX were included. This suggests that AL may be associated with nurses’ innovation primarily through psychological and relational mechanisms rather than through a direct pathway. More importantly, PE represented the stronger pathway. Its indirect effect was significantly larger than that of LMX, accounting for approximately 60.6% of the total effect compared with 30.8% for LMX. This finding indicates that AL may promote innovation primarily by strengthening nurses’ sense of meaning, competence, self‐determination and impact, while high‐quality leader–member relationships provide an additional relational mechanism.

### 5.2. AL as a Distal Leadership Resource for Innovation

AL was strongly associated with both PE and LMX, supporting the view that authentic leaders create conditions that enable innovation rather than directly producing IB. Authentic leaders demonstrate transparency, ethical consistency and balanced decision‐making, which help employees interpret their work environment as trustworthy, supportive and meaningful [4]. These leadership cues may encourage nurses to perceive greater autonomy and influence over their work, while simultaneously strengthening relational trust with leaders.

A particularly noteworthy finding was that the direct association between AL and nurses’ IB became nonsignificant after PE and LMX were included in the model. Although previous studies have reported positive associations between AL and IB [5, 28], many of these studies relied on cross‐sectional designs and focused primarily on direct effects. The present findings suggest that AL may function as a distal leadership resource whose influence is transmitted through psychological and relational mechanisms rather than through a direct pathway.

This interpretation is consistent with SIPT and SET. Leadership behaviours shape how employees interpret their work environment and how they experience workplace relationships [10, 11]. Consequently, AL appears to foster innovation not by directly stimulating innovative actions but by creating the psychological and relational conditions that make innovation more likely.

### 5.3. Psychological Empowerment as the Primary Cognitive–Motivational Pathway

PE emerged as the strongest mediating pathway linking AL and nurses’ IB. This finding supports SIPT, which proposes that employees rely on cues from their social environment to construct perceptions that guide subsequent behaviour [10]. Authentic leaders may signal trust, respect and confidence in nurses’ abilities, thereby strengthening nurses’ perceptions of meaning, competence, self‐determination and impact [6].

The stronger indirect effect observed through PE is theoretically important. IB is inherently proactive and discretionary, requiring employees to identify opportunities for improvement, challenge existing routines and persist despite uncertainty or potential failure. Such behaviours are unlikely to occur unless employees believe that they are capable of making meaningful contributions and that their actions can influence organisational outcomes. Consistent with previous research, PE has been identified as a key motivational mechanism through which leadership influences innovation‐related outcomes [12, 29].

Importantly, PE accounted for approximately 60.6% of the total effect, significantly exceeding the contribution of LMX. This finding extends existing nursing leadership research by suggesting that AL promotes innovation primarily through enhancing nurses’ internal psychological resources rather than solely through strengthening interpersonal relationships. In highly regulated healthcare environments, nurses may require a strong sense of autonomy, competence and impact before they are willing to engage in innovation activities that extend beyond routine clinical responsibilities.

### 5.4. LMX as a Complementary Relational Pathway

LMX also significantly mediated the relationship between AL and nurses’ IB, supporting SET. According to this perspective, employees who experience supportive and trusting relationships with leaders are more likely to reciprocate through discretionary behaviours that benefit the organisation [8, 11]. Authentic leaders may strengthen LMX by demonstrating fairness, openness and consistency, thereby fostering mutual trust and respect.

This finding is consistent with previous studies demonstrating positive relationships between LMX and IB [29, 30]. High‐quality LMX relationships may provide nurses with emotional support, access to resources and psychological safety, all of which are important for innovation. Nurses who trust their leaders may be more willing to voice new ideas, experiment with improvements and accept the risks associated with innovation.

However, the indirect effect through LMX was significantly smaller than that through PE. This suggests that relational support alone may not be sufficient to facilitate innovation unless nurses also perceive themselves as capable, autonomous and influential. LMX may, therefore, function as a complementary relational mechanism that facilitates innovation by providing support and encouragement, while PE serves as the primary motivational driver of IB.

### 5.5. Theoretical Contribution of the Parallel Mediation Framework

A major contribution of this study is the integration of SIPT and SET within a single parallel mediation framework. Previous studies examining nurses’ IB have often focused on isolated leadership variables or single mediating mechanisms [29, 31]. Such approaches provide limited insight into how leadership effects are translated into innovation within complex healthcare environments.

The present findings demonstrate that AL influences IB through two distinct yet complementary mechanisms. PE represents a cognitive–motivational pathway through which nurses interpret leadership cues and develop the confidence and autonomy necessary for innovation. LMX represents a relational pathway through which nurses experience support, trust and reciprocal obligations toward their leaders. By examining these mechanisms simultaneously, the study moves beyond identifying whether AL is associated with innovation and instead clarifies how such influence occurs.

Furthermore, the significant difference between the two indirect effects refines existing leadership theories by showing that the cognitive–motivational pathway was substantially stronger than the relational pathway. This finding suggests that, within nursing contexts, AL may promote innovation primarily by strengthening nurses’ internal psychological resources, while relational exchanges play a supportive but secondary role. In doing so, the study contributes to a more nuanced and mechanism‐focused understanding of leadership and innovation in healthcare organisations.

### 5.6. Implications for Nursing Management

The findings have several implications for nursing management. First, leadership development programmes should not only teach AL behaviours in general but should explicitly focus on how these behaviours enhance nurses’ PE. Nurse managers can support empowerment by involving nurses in decision‐making, recognising their contributions, granting appropriate autonomy and helping them see the impact of their work on patient care and service improvement.

Second, because PE was the stronger pathway, organisations seeking to promote innovation should prioritise empowerment‐oriented management practices. These may include protected time for improvement activities, opportunities for nurses to lead small‐scale innovation projects, recognition of practice‐based improvements and feedback systems that show how nurses’ ideas influence care processes.

Third, high‐quality LMX remains important. Nurse managers should foster trust, respect and individualised support so that nurses feel safe to share ideas and engage in innovation‐related activities. However, relational support should be combined with empowerment strategies. A supportive relationship may encourage nurses to participate, but empowerment may determine whether they feel capable of initiating and sustaining innovation.

### 5.7. Strengths, Limitations and Future Research

This study has several strengths, including a large multicentre sample, a two‐wave time‐lagged design and the use of SEM to test parallel mediation mechanisms while accounting for measurement error. The inclusion of the direct path from AL to IB also allowed a more rigorous assessment of whether the association was direct or fully mediated.

Several limitations should be considered. First, although the two‐wave design introduced temporal separation and reduced the risk of common method bias, it does not establish definitive causality. Second, all variables were self‐reported, which may introduce perceptual bias. Future studies could incorporate supervisor ratings, objective innovation indicators or multisource assessments. Third, the 1‐month interval was selected to balance temporal separation and participant retention, but alternative time intervals may produce different estimates of leadership effects. Although the retention rate was acceptable for a two‐wave survey design, attrition may have introduced potential selection bias. Future studies should further examine whether longitudinal patterns differ among participants with different follow‐up completion characteristics. Fourth, the sample was drawn from public hospitals in China, which may limit transferability to other healthcare systems or cultural contexts.

Future research should examine whether the relative strength of PE and LMX varies across organisational climates, leadership levels, hospital types or cultural settings. Intervention studies are also needed to determine whether strengthening AL and PE can produce sustained improvements in nurses’ IB.

## 6. Conclusion

This study provides empirical evidence that AL is associated with nurses’ IB through two complementary mechanisms: PE and LMX. By integrating SIPT and SET within a parallel mediation framework, the study advances understanding of how AL fosters innovation in nursing practice.

The findings indicate that AL does not directly influence nurses’ IB. Instead, its effects are transmitted through psychological and relational processes. PE emerged as the stronger pathway, suggesting that AL primarily promotes innovation by enhancing nurses’ sense of meaning, competence, self‐determination and impact, while LMX serves as a complementary relational mechanism.

These findings contribute to nursing leadership research by moving beyond single‐mechanism explanations and providing a more nuanced understanding of how leadership influences innovation in complex healthcare environments. For nursing management, the results suggest that efforts to foster innovation should prioritise strategies that strengthen nurses’ PE while simultaneously cultivating high‐quality leader–member relationships. AL may, therefore, represent an important leadership resource for healthcare organisations seeking to support sustainable innovation in clinical practice.

**Nomenclature**
AL: Authentic leadership
PE: Psychological empowerment
LMX: Leader–member exchange
IB: Nurses’ innovative behaviour
SEM: Structural equation modelling
CFA: Confirmatory factor analysis
CR: Composite reliability
AVE: Average variance extracted

## Author Contributions

Gui Yu, Jing Fang and Xiaojuan Yang contributed equally to this work. Gui Yu contributed to conceptualisation, data curation, formal analysis, visualisation and writing–original draft. Jing Fang and Xiaojuan Yang contributed to data curation, formal analysis, investigation, visualisation and writing–original draft. Lijuan Yin contributed to investigation, project administration and supervision. Yanli Zeng contributed to conceptualisation, methodology, formal analysis, statistical interpretation, supervision, project administration and writing–review and editing.

## Funding

This research received no specific grant from any funding agency in the public, commercial or not‐for‐profit sectors.

## Conflicts of Interest

The authors declare no conflicts of interest.

## Supporting Information

Additional supporting information can be found online in the Supporting Information section.

## Supporting information


**Supporting Information** Supporting Table S1. Differences in authentic leadership, psychological empowerment and leader–member exchange according to demographic characteristics.

## Data Availability

The data that support the findings of this study are available from the corresponding author upon reasonable request.
