# Supplementary material for: Authentic Leadership and Nurses’ Innovative Behaviour: A Two‐Wave Multicentre Study of Psychological Empowerment and Leader–Member Exchange
Source: J Nurs Manag. 2026 Aug 30;2026:7337513. doi: 10.1155/jonm/7337513 (PMC13527385; doi:10.1155/jonm/7337513)
Supplement: Supplementary file 1 [file JONM-2026-7337513-s001.docx]

Supplementary Table S1. Differences in authentic leadership, psychological empowerment, and leader–member exchange according to demographic characteristics

| **Demographic characteristics** | **AL** | **PE** | **LMX** |
| --- | --- | --- | --- |
| Gender | *t* = -0.437, *p* = .663 | *t* = 0.990, *p* = .324 | *t* = 0.888, *p* = .376 |
| Age group | *F* = 6.212, *p* < .001 | *F* = 5.215, *p* = .001 | *F* = 3.147, *p* = .024 |
| Education level | *F* = 2.289, *p* = .102 | *F* = 2.289, *p* = .102 | *F* = 1.627, *p* = .197 |
| Work experience | *F* = 9.828, *p* < .001 | *F* = 0.722, *p* = .486 | *F* = 6.690, *p* = .001 |
| Hospital level | *F* = 0.331, *p* = .857 | *F* = 1.044, *p* = .383 | *F* = 0.612, *p* = .654 |

Note. AL = authentic leadership; PE = psychological empowerment; LMX = leader–member exchange. Independent-sample t-tests were used for two-group comparisons, and one-way analyses of variance were used for comparisons among multiple groups.
